# Application of FTIR-ATR Spectroscopy to Determine the Extent of Lipid Peroxidation in Plasma during Haemodialysis

**DOI:** 10.1155/2015/245607

**Published:** 2015-04-19

**Authors:** Adam Oleszko, Sylwia Olsztyńska-Janus, Tomasz Walski, Karolina Grzeszczuk-Kuć, Jolanta Bujok, Katarzyna Gałecka, Albert Czerski, Wojciech Witkiewicz, Małgorzata Komorowska

**Affiliations:** ^1^Institute of Biomedical Engineering and Instrumentation, Faculty of Fundamental Problems of Technology, Wrocław University of Technology, Wybrzeże Wyspiańskiego 27, 50-370 Wrocław, Poland; ^2^Regional Specialist Hospital in Wrocław, Research and Development Centre, Kamieńskiego 73a, 51-124 Wrocław, Poland; ^3^Department of Animal Physiology and Biostructure, Faculty of Veterinary Medicine, Wrocław University of Environmental and Life Sciences, Norwida 31, 50-375 Wrocław, Poland

## Abstract

During a haemodialysis (HD), because of the contact of blood with the surface of the dialyser, the immune system becomes activated and reactive oxygen species (ROS) are released into plasma. Particularly exposed to the ROS are lipids and proteins contained in plasma, which undergo peroxidation. The main breakdown product of oxidized lipids is the malondialdehyde (MDA). A common method for measuring the concentration of MDA is a thiobarbituric acid reactive substances (TBARS) method. Despite the formation of MDA in plasma during HD, its concentration decreases because it is removed from the blood in the dialyser. Therefore, this research proposes the Fourier Transform Infrared Attenuated Total Reflectance (FTIR-ATR) spectroscopy, which enables determination of primary peroxidation products. We examined the influence of the amount of hydrogen peroxide added to lipid suspension that was earlier extracted from plasma specimen on lipid peroxidation with use of TBARS and FTIR-ATR methods. Linear correlation between these methods was shown. The proposed method was effective during the evaluation of changes in the extent of lipid peroxidation in plasma during a haemodialysis in sheep. A measurement using the FTIR-ATR showed an increase in plasma lipid peroxidation after 15 and 240 minutes of treatment, while the TBARS concentration was respectively lower.

## 1. Introduction

Dialysis therapy allows for the removal of metabolic waste products and excess water and electrolytes from the bloodstream of patients with renal failure. The treatment is performed by putting the patient's blood through the dialyser where it comes into contact with a semipermeable membrane with dialysis fluid, the content of which allows for regeneration of the blood buffer system.

Contact of the patient's blood with surfaces of drains and the dialyser leads to activation of the immune system [1], resulting in intensified production of reactive oxygen species (ROS) via the activation of neutrophils and platelets—a so-called “oxidative burst” [2]. Apart from the super oxide radical, one ROS whose concentration rises during an oxidative burst is hydrogen peroxide. The ROS present in the blood causes oxidation of the compounds contained therein. Plasma lipids and phospholipids, being part of the erythrocyte cell membrane, are particularly susceptible to ROS activity [1].

Research conducted by Haklar et al. [3] confirms the presence of a lipid peroxidation process during haemodialysis. After the treatment, an increase in the quantity of conjugated dienes from 3.75 to 5.31 nmol per 1 *μ*mol of lipids extracted from plasma was observed. It has also been proven that, during haemodialysis, a peroxidation of cholesterol from the cell membranes of erythrocytes [4] and a decrease in the concentration of antioxidants in plasma [5] take place.

Lipid peroxidation is associated with the formation of peroxides as primary products [6]. They are broken down into secondary products with shorter hydrocarbon chains. One such product is malondialdehyde (MDA). There are methods that allow to determine the content of both primary and secondary products [7].

One method of testing the primary products of oxidation is the method of iodometric titration. It is based on the oxidation of iodide ions (*I* ^−^) by lipid peroxides. A standard solution of potassium iodide is added to the lipid specimen. As a result of the reaction, molecular iodine (*I*_2_) is produced, which is then titrated with a standard sodium thiosulphate solution in the presence of starch [8].

Another method for testing primary products of lipid oxidation is based on the detection of conjugated dienes. Their UV-VIS spectra are characterised by the presence of an ultraviolet absorption band (230–235 nm). The greater the oxidation of fatty acids in the specimen, the greater the absorbance [9].

MDA is one of the breakdown products of lipid peroxides; thus, it constitutes a secondary oxidation product. Determination of its concentration is used as an indicator of lipid oxidation. MDA is used in a reaction with thiobarbituric acid (TBA), during which a coloured MDA-TBA complex is formed, absorbing at 530–535 nm [10]. The disadvantage of this method is its low specificity. Chemical compounds other than MDA that are present in biological specimens can react with TBA, for example, bilirubin, sialic acid (found in cell membranes), degradation products of carbohydrates, and other aldehydes [11, 12]. Therefore, it is more about determining the quantity of products that are reactive with TBA (thiobarbituric acid reactive substances, TBARS). Nevertheless, determining the concentration of MDA is one of the most popular methods for studying lipid oxidation and oxidative stress magnitude.

A limitation of this method for studying the effects of haemodialysis on plasma lipid peroxidation is the removal of MDA from plasma that takes place on the dialyser during treatment. Therefore, despite the increased formation of MDA in plasma during treatment, its concentration after dialysis is lower than before the patient was connected to the dialysis machine [13, 14]. This makes it impossible to apply the method of determining TBARS to study the effects of haemodialysis on plasma lipid peroxidation. The solution to this problem was supposed to be a commercial, fast assay kit for measuring ROS metabolites named the d-ROMs test (Diacron International, Grosseto, Italy) [15]. However, this method is controversial in relation to the possibility of interference of ceruloplasmin on the value of the obtained result [16, 17].

An FTIR spectroscopy allows for a direct observation of the appearance or disappearance of bands that come from distinct vibrations of functional lipid groups, whose changes indicate a formation of primary oxidation products [18–20]. One of these bands is stretching vibration of C=O group. Increase of *ν*(C=O) band is result of three-step peroxidation process, which undergoes according to following scheme. Firstly, during stage of initiation, lipid molecule (LH) reacts with ROS. Transfer of hydrogen from lipid to ROS and appearance of lipid radical (L^•^) are effects of this reaction (1). Secondly, lipid radical undergoes reaction with molecular oxygen (2a), giving lipid superoxide radical molecule (LOO^•^), which is able to react with another lipid molecule (2b).

Initiation:

(1) $$\begin{array}{c} \mathrm{LH}+\;\text{ROS}⟶\text{ROSH}+{\text{L}}^{•} \end{array}$$

Propagation: 
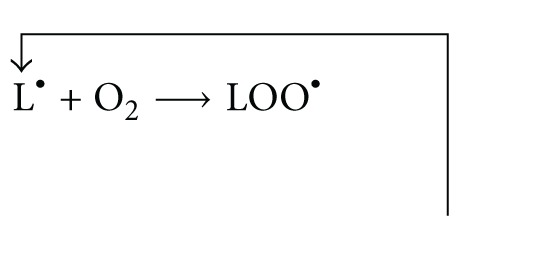
(2a)

(2b) $$\begin{array}{c} {\text{LOO}}^{•}+\mathrm{LH}⟶{\text{LOOH}+\text{L}}^{•} \end{array}$$

Termination:

(3a) $$\begin{array}{c} {\text{L}}^{•}+{\text{L}}^{•}⟶\text{L-L} \end{array}$$

(3b) $$\begin{array}{c} {\text{LOO}}^{•}+{\text{LOO}}^{•}⟶\text{L=O}+\text{LOH}+{\text{O}}_{2} \end{array}$$

(3c) $$\begin{array}{c} {\text{LOO}}^{•}+{\text{L}}^{•}⟶\text{L=O}+\text{LOH} \end{array}$$

Generation of lipid superoxide molecule (LOOH) and regeneration of lipid radical (which can react with another oxygen and lipid molecule) are results of propagation stage. Regeneration of lipid radical shows chain reaction character of peroxidation, which can be terminated only during reaction of two radical molecules. Lipid dimers (L-L), hydroxides (LOH), and oxides (L=O) belong to products of termination stage. Products of reactions (3b) and (3c) (lipid oxides) are responsible for increase of absorbance of *ν*(C=O) band.

The aim of our experiments was to test the usefulness of infrared spectroscopy with a Fourier transform, utilising the attenuated total reflectance (FTIR-ATR), to assess the extent of lipid peroxidation in the plasma of dialysed patients.* In vitro* model tests were performed by oxidising total plasma lipid fractions using H_2_O_2_. Concurrently, the FTIR-ATR spectra were recorded, and the degree of lipid peroxidation was determined using the classic TBARS method, looking for a correlation between the two methods. Analysis of the FTIR-ATR spectra allowed us to define the bands corresponding to the peroxidation process. The developed procedure was used for the analysis of plasma taken from the blood of dialysed sheep and for determination of change in the degree of lipid peroxidation during treatment.

## 2. Materials and Methods 

### 2.1. Lipid Oxidation in Aqueous Suspension

#### 2.1.1. Plasma

Blood specimens of 20 mL in volume were collected from five clinically healthy male sheep (rams of the Polish Merino breed) with a body mass from 50 to 60 kg. A total of 100 mL of blood was collected. To prevent clotting, 3.8% of disodium citrate (POCH S.A., Poland) was added to the blood specimen. The volume ratio of blood to anticoagulant was 9 : 1.

Because of the need to transport blood specimens from the collection site to a laboratory within one hour after collection, blood specimens were subjected to centrifugation for 10 minutes at 3000 rpm after which plasma was collected for further testing.

#### 2.1.2. Lipid Extraction

Lipid extraction was performed separately for each blood specimen based on a method developed by Folch et al. [21]. For this purpose, a mixture of chloroform RG (POCH S.A., Poland) with methanol RG (POCH S.A., Poland) at a ratio of 2 : 1 was prepared. After this, a mixture of chloroform-methanol and plasma in a volume ratio of 9 : 1 was added to the tubes. The tubes content was shaken for five minutes. Next, most of the protein precipitate was removed from the mixture through filtration.

In order to remove the rest of the proteins from the supernatant, a small amount of deionised water in the ratio of 1 : 10 was added. Again, it was shaken for five minutes and centrifuged for 10 minutes at 3800 rpm (centrifuge MPW-350R, MPW MED. Instruments, Poland). As a result of centrifugation, a separation into two phases followed: an upper (aqueous) containing protein and a lower (organic) containing lipid fraction. The upper phase together with the rest of the protein precipitate was removed, after which the procedure of removing proteins was repeated.

Lipids from all specimens that were contained in the isolated organic phase were mixed. The solvent was evaporated in vacuum. After evaporation, approximately 160 mg of extracted lipids remained in the dish.

#### 2.1.3. Preparation of Aqueous Lipid Suspension

A suspension of multilamellar liposomes was prepared from the extracted lipids for further oxidation. After evaporation of the solvent, deionised water was added to the dry weight and sonicated until a milky white suspension was obtained. Lipid concentration amounted to 1.0 mg/mL.

#### 2.1.4. Oxidation of Lipids in a Suspension Using Hydrogen Peroxide

Hydrogen peroxide, H_2_O_2_, with a 30% concentration (POCH S.A., Poland) was prediluted to 0.5%.

An aqueous lipid suspension with a concentration of 1 mg/mL was oxidised using H_2_O_2_. The concentrations of hydrogen peroxide that were used were 0, 0.50, 2.50, 10, and 20 mM. This procedure (repeated 3 times) allowed for a selection of oxidant concentrations in the subsequent part of the experiment: 0 (control group), 0.25, 0.50, 0.75, and 1.0 mM. The samples of lipid suspension were incubated in a thermomixer at 37°C for 30 min. Then, each sample was divided into two parts. The first part, with a volume of 1.5 mL, was intended to determine the concentration of TBARS. The second part with a volume of 2.0 mL was intended for testing by means of the FTIR-ATR spectroscopy. The experiment was repeated seven times for both methods (*N* = 7).

#### 2.1.5. Determining the TBARS Concentration

To determine the TBARS concentration, a 0.37% solution of thiobarbituric acid TBA (AppliChem, Germany) in a 0.25 HCl molar solution (Chempur, Poland) was used in the test specimen.

A suspension of oxidised lipids was mixed with a TBA solution in the ratio of 1 : 1 and incubated in a water bath at 100°C for 10 minutes. Concurrently, a reference specimen was prepared (a mixture of deionised water and TBA solution in the ratio of 1 : 1). After incubation, the cooled specimens were transferred to cuvettes and, with the use of a spectrophotometer UV-VIS Evolution 60S (Thermo Scientific, USA), the absorbance at a wavelength of 535 and 600 nm was measured with respect to the reference specimen. The absorbance difference (*A*_diff_ = *A*_535_ − *A*_600_) is a measure of the concentration of TBARS in a specimen [22].

Next, a change in the value of the *A*_diff_ parameter in relation to the lipid control specimen (*A*_0_), not subjected to oxidation (H_2_O_2_ concentration equal to 0 mM), was determined according to

(4) $$\begin{array}{c} \Delta_{\text{TBARS}}=\frac{A_{\text{diff}}-A_{0}}{A_{0}}\cdot 100\%. \end{array}$$

A mean value of the parameter Δ_TBARS_ and standard deviation were determined for each value of H_2_O_2_ concentration from each seven measurement series.

#### 2.1.6. Making Plasma Lipid Films on an ATR Accessory

**Figure 1:**
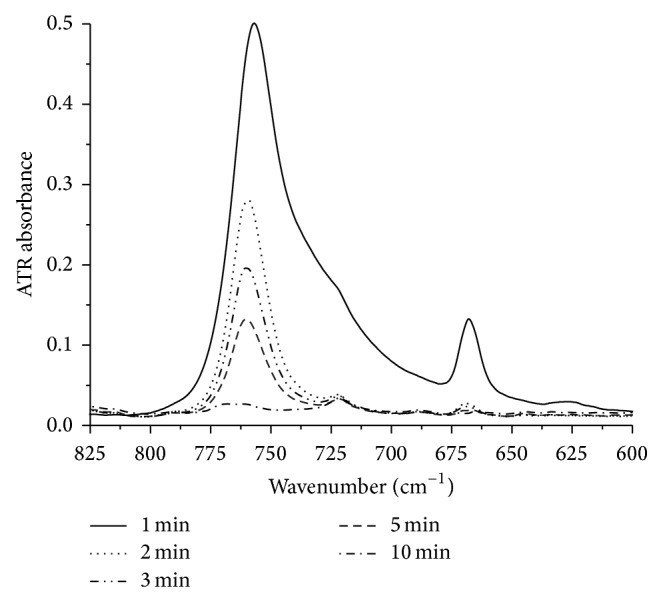
ATR-FTIR spectra of phospholipids dissolved in chloroform; a decrease in band absorbance derived from chloroform at 756 and 668 cm^−1^ during the formation of film.

A specimen of the aqueous plasma lipid suspension with a volume of 2 mL, prepared as described in Section 2.1.4, was added with a 3 mL mix of chloroform-methanol and then shaken for five minutes in order to extract the lipids from the aqueous phase. After centrifugation of the specimens for 10 minutes at 3800 rpm, the organic phase was separated from the aqueous phase. The organic phase was concentrated via evaporation of the lipid solution in the mixture to a volume of about 50 *μ*L. From the remaining volume, after concentration, 20 *μ*L were taken out and placed on a crystal of the ATR sampling accessory and formed film. During the evaporation of the solvent, the FTIR-ATR spectra were recorded-up to a disappearance of the band at 760 cm^−1^, indicating the presence of chloroform (Figure 1). The last spectrum was recorded two minutes after evaporation of the solvent and used for further analysis.

#### 2.1.7. A Test Using the FTIR Spectroscopy

Spectroscopic tests were performed using a spectrophotometer FTIR Nicolet 6700 (Thermo Scientific, USA) with an ATR accessory with a diamond crystal. The recorded spectra were in the form of means of 32 spectra, performed in the 4000–400 cm^−1^ range with a 4 cm^−1^ resolution and atmospheric correction switched on at room temperature (25°C).

Based on the literature [15–17] and own research, two bands were selected for analysis, *ν*_as_(CH_3_) at 2958 cm^−1^ and *ν*(C=O) at 1738 cm^−1^, corresponding to a stretching vibration of the CH_3_ group and a stretching vibration of the C=O group. Together with lipid oxidation, there is an increase of integral absorbance of the band *ν*(C=O).

Values of integral absorbance of the bands *ν*_as_(CH_3_) in range 2982–2942 cm^−1^ and *ν*(C=O) in range 1787–1685 cm^−1^ were calculated using the programme GRAMS AI/IQ (parameters *A*_2958_ and *A*_1738_). In order to standardise the results, the ratio of the integral absorbance of the band *ν*(C=O) with respect to the band *ν*_as_(CH_3_) was calculated according to

(5) $$\begin{array}{c} I=\frac{A_{1738}}{A_{2958}}. \end{array}$$

Next, the change in the value of parameter *I* was determined in relation to the control specimen (*I*_contr_) not subjected to oxidation (H_2_O_2_ concentration equal to 0 mM), according to the formula below:

(6) $$\begin{array}{c} \Delta I=\frac{I-I_{\text{contr}}}{I_{\text{contr}}}\cdot 100\%. \end{array}$$

A mean value of the parameter Δ*I* and standard deviation were determined for each value of the H_2_O_2_ concentration from each seven measurement series.

### 2.2. Degree of Lipid Peroxidation in the Process of Haemodialysis

#### 2.2.1. Haemodialysis Treatment Procedure

Experiments were performed on a clinically healthy adult male sheep (ram of the Polish Merino breed) weighing 56 ± 2 kg. Vascular access was obtained by percutaneous placement of 14.5 Fr × 28 cm dual-lumen Hemo-Flow Dialysis Catheter (Medcomp, USA) into the right jugular vein (vena jugularis) under premedication (xylazine 0.2 mg/kg body weight intravenously) and local anaesthesia by infiltration (2% lidocaine).

The experiments were performed using the Fresenius 4008B haemodialysis machine on a sheep without pharmacological taming standing in a pit. The animal was subjected to dialysis treatments lasting four hours, three times a week using the Fresenius F4HPS polysulphone dialyser and standard bloodlines (AV-Set, Fresenius, Poland). Before the treatment, 5000 IU of heparin sodium (WZF Polfa S.A., Poland) was administered in a 500 mL Ringer's solution (B. Braun, Germany) into the drip infusion. During the experiment, heparin (at a dose of 1250 IU/h) was administered into the bloodline. The dosage of heparin was established in preliminary experiments. During four hours of haemodialysis, 36 l of blood was circulated through an extracorporeal set (blood flow rate: 150 mL/min), the total ultrafiltration was 200 mL. The flow rate of dialysate (composition: Na^+^ 142.0; K^+^ 3.0; Mg^2+^ 0.5; Ca^2+^ 1.5; Cl^−^ 110.0; HCO^3−^ 32.0 mmol/L; glucose 1.0 g/L) was preset to 500 mL/min. Five haemodialysis treatments were performed as part of this experiment.

#### 2.2.2. Sampling

During each haemodialysis, three blood specimens of 6 mL in volume were taken using disodium citrate as described in Section 2.1.1. Blood specimens were taken from the venous line. The first specimen was taken immediately after commencement of treatment, and the second was taken 15 minutes from the start of treatment. The time of specimen collection was based on the literature [23]; according to which, the greatest changes in blood are observed during the first 30 minutes of haemodialysis. The third specimen was taken just before the end of the circulation, after 240 minutes.

#### 2.2.3. Determining the TBARS Concentration in Plasma

To determine the TBARS concentration, a 0.37% solution of thiobarbituric acid TBA (AppliChem, Germany) was used in a 0.25 HCl molar solution (Chempur, Poland) and 15% trichloroacetic acid solution TCA (Chempur, Poland) in a 0.25 HCl molar solution.

To 0.5 mL of plasma we added 0.5 mL of TCA. Specimens were subjected to one hour of incubation at 4°C then centrifuged for 20 minutes at 4500 rpm; after which, 0.5 mL of supernatant was collected. Next, 0.5 mL of TBA was added to the supernatant; after which, the specimens were incubated in a water bath at 100°C for 10 minutes. Concurrently, a reference specimen was prepared (deionised water, a solution of TBA and TCA in the ratio of 1 : 1 : 1). A measurement of TBA using a spectrophotometer was performed in a manner described in Section 2.1.5.

#### 2.2.4. Extraction of Plasma Lipids and a Test Using the FTIR-ATR Spectroscopy

Lipids were extracted from 3 mL of plasma for each one of the specimens as described in Sections 2.1.1 and 2.1.2. After placing the specimen on a crystal, its spectrum was recorded until disappearance of the band at 760 cm^−1^. Recording of the spectra and its analysis was conducted in a manner described in Sections 2.1.6 and 2.1.7.

#### 2.2.5. Statistical Analysis

For data analysis, Matlab R2009b software by MathWorks (USA) was used. The results were presented in the form of mean values and standard deviations. The differences between particular parameters were tested using the parametric ANOVA test. The threshold of statistical significance was set at *P* < 0.05.

#### 2.2.6. Ethics Committee Approval

In order to conduct the experiments, authorisation was obtained in accordance with the resolution number 10/2013 of the II Local Ethics Committee in Wrocław, regarding experiments on animals.

## 3. Results

### 3.1. Results of Lipid Oxidation in Aqueous Suspension

#### 3.1.1. FTIR-ATR Spectroscopy

Figure 1 shows FTIR-ATR spectra of plasma lipids dissolved in chloroform. The decrease in band absorbance at 760 cm^−1^ is related to the evaporation of chloroform after placing the specimen on a diamond crystal. All analyses were performed for spectra recorded after the evaporation of chloroform.

**Figure 2:**
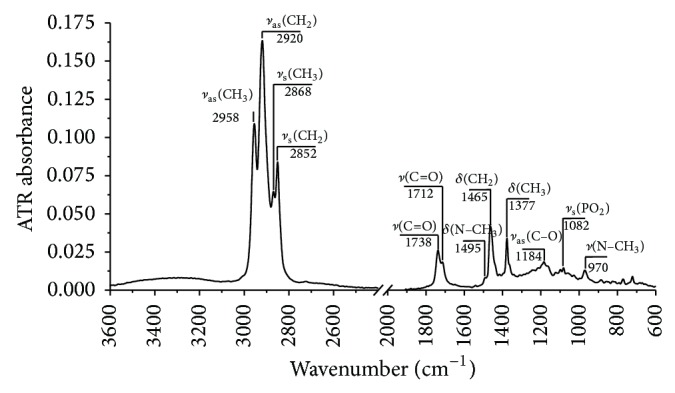
FTIR-ATR spectrum of extracted plasma lipids after evaporation of chloroform.

Figure 2 shows the FTIR-ATR spectrum of lipids extracted from plasma. A description of the assigned absorption bands is listed in Table 1. In range from 3050 to 2750 cm^−1^, the bands correspond to asymmetric and symmetric stretching vibrations of the methyl and methylene groups [24–32]. The asymmetric and symmetric bands of the methyl groups were labelled as *ν*_as_(CH_3_) and *ν*_s_(CH_3_), respectively. Their maxima occur at wavenumbers 2958 and 2868 cm^−1^, whereas asymmetric and symmetric bands of methylene groups labelled as *ν*_as_(CH_2_) and *ν*_s_(CH_2_) occur at 2920 and 2852 cm^−1^.

At 1738 cm^−1^, there is a very important band associated with stretching vibrations of the carbonyl group *ν*(C=O): particularly ester bonds between fatty acids and glycerol within the lipid molecules. However, this type of bond may also be formed by peroxidation of fatty acid chains [34]. Therefore, we suggest that the increase in the intensity of this band indicates an increase in lipid oxidation in the specimen, which has been confirmed in the literature [25–30, 32, 35].

Subsequent bands are located at the following wave numbers: 1495 cm^−1^ (a stretching vibration of the N–CH_3_ group derived from choline), 1465 and 1377 cm^−1^ (bending vibrations of the methyl and methylene groups of fatty acids), 1184 cm^−1^ (a stretching vibration of the C–O group of lipid ester bond), and 1082 cm^−1^ (a stretching vibration of the phosphate PO_2_ group) [24–29, 33].

**Figure 3:**
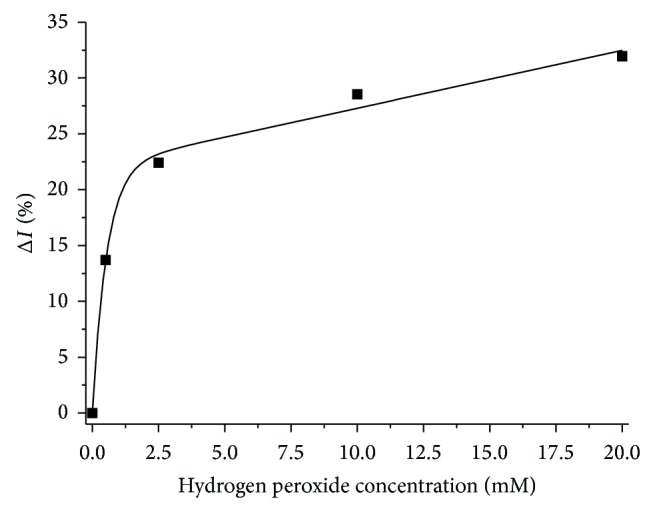
Dependence of change in the ratio of integral band absorbance *ν*(C=O) with respect to *ν*_as_(CH_3_); upon the concentration of H_2_O_2_ in the concentration range from 0 to 20 mM; mean value relative to control sample (*N* = 3).

In the beginning, the dependence of the value of parameter Δ*I* upon the H_2_O_2_ concentration function was calculated (Figure 3). This relationship is exponential in nature, taking into account the whole range of H_2_O_2_ concentrations (0–20 mM). In some range, exponential function can be approximated by linear function. We assume, that the above mentioned ratio can be described by linear, rapid growth up to the value of 1.0 mM. This reflects an increase in the oxidation of lipids extracted from plasma in the H_2_O_2_ concentration function. Therefore, subsequent experiments were performed for hydrogen peroxide with a concentration ranging from 0–1.0 mM.

**Figure 4:**
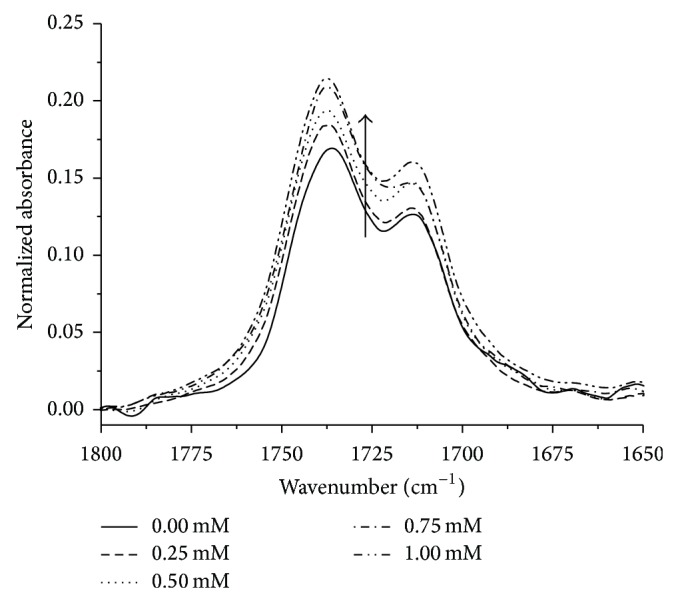
FTIR-ATR spectra of lipid film, corresponding to the band *ν*(C=O); increase in band absorbance due to H_2_O_2_.

**Figure 5:**
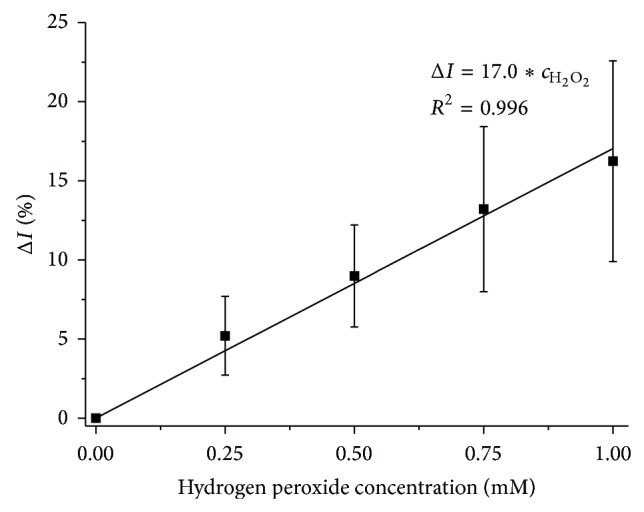
Dependence of changes in the ratio of integral band absorbance *ν*(C=O) with respect to *ν*_as_(CH_3_) upon the concentration of H_2_O_2_; mean value relative to control sample ± standard deviation (*N* = 7).

Figure 4 shows spectra from one series of measurement of plasma lipid oxidation in the spectral range 1800–1650 cm^−1^ with band *ν*(C=O). Each of the spectra have been normalised with respect to band *ν*_as_(CH_3_). The increase in the intensity of band *ν*(C=O) as a result of oxidation is visible. The linear increase in the value of the parameter Δ*I* in the H_2_O_2_ concentration function (Figure 5) indicates that lipid oxidation is taking place.

#### 3.1.2. Determination of TBARS

**Figure 6:**
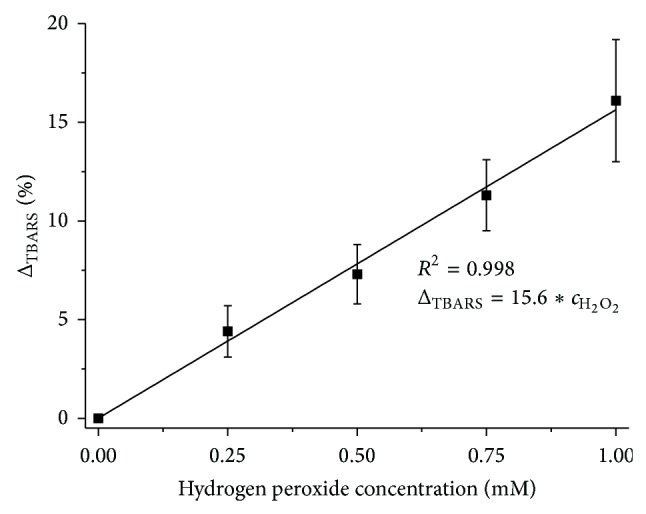
Dependence of changes in TBARS concentration in a sample upon the concentration of H_2_O_2_; mean value relative to control sample ± standard deviation (*N* = 7).

A linear change in the TBARS concentrations has been observed relative to the control specimen when H_2_O_2_ was added. The results are shown in Figure 6. Therefore, it can be ascertained that the amount of TBARS that were formed is proportional to the amount of agent that causes oxidation in the H_2_O_2_ concentration ranges up to 1 mM and has a fixed lipid concentration level in the specimen. Smaller values of standard deviations were recorded than in the case of using FTIR-ATR spectroscopy.

### 3.2. Lipid Peroxidation under the Influence of Haemodialysis

Figure 8 illustrates the changes in the amount of peroxidation products during haemodialysis. A statistically significant increase (*P* < 0.05) was found in the amount of lipid peroxidation products of plasma after 15 and 240 minutes of treatment relative to the control specimen, which was determined by using FTIR-ATR spectroscopy. This increase amounted to 44% and 29%, respectively.

In the case of determining TBARS, a decrease of 15% and 41% was recorded, respectively, after 15 and 240 minutes of haemodialysis; only the decrease after 240 minutes was statistically significant (*P* parameter was 0.074 and 0.006, resp.).

## 4. Conclusions

**Figure 7:**
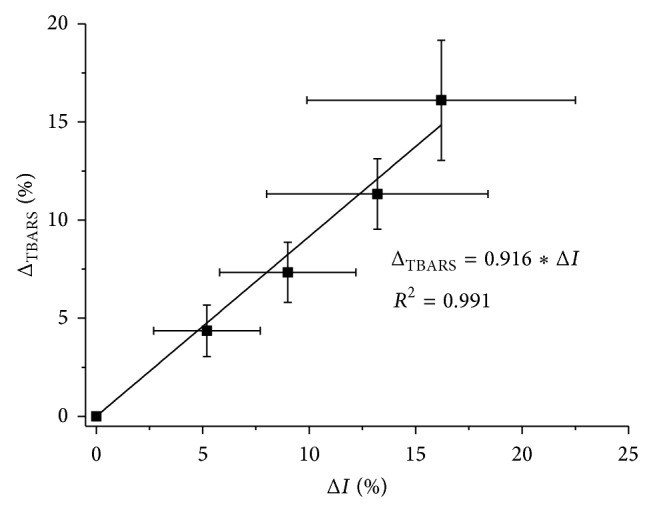
Correlation between change of the ratio of integral band absorbance *ν*(C=O) with respect to *ν*_as_(CH_3_) and change in the concentration of TBARS; mean value relative to control sample ± standard deviation (*N* = 7).

**Figure 8:**
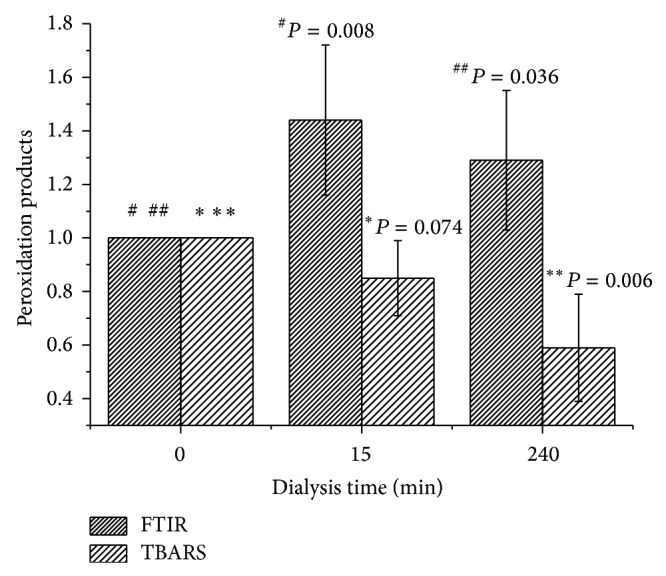
Dependence of the amount of peroxidation products relative to control sample (0 min HD) upon duration of the haemodialysis treatment determined by using FTIR-ATR spectroscopy and TBARS (*N* = 5).

During* in vitro* model tests, a relationship was confirmed between the value of the ratio of absorbance bands *ν*(C=O) with respect to *ν*_as_(CH_3_) and concentration of TBARS and the amount of H_2_O_2_ added to lipid specimen extracted from plasma. A correlation between the results obtained using FTIR spectroscopy and results of the TBARS method was investigated (Figure 7). The results obtained by both methods show high consistency. Despite the fact that FTIR-ATR spectroscopy investigated primary products of lipid oxidation and the determination of TBARS method is based on detection of its breakdown products (secondary oxidation products), the results are comparable.

Although FTIR-ATR spectroscopy showed lower reproducibility between different series of measurements than the TBARS method, which resulted in high standard deviation values, the lipid peroxidation test technique using FTIR-ATR spectroscopy allowed for the determination of oxidative stress in plasma during haemodialysis, while the method for determining TBARS concentrations showed that MDA is effectively removed by the dialyser membranes.

Determination of TBARS showed a decrease in the amount of secondary peroxidation products during haemodialysis by 15% and 41% after 15 and 240 minutes as a result of MDA's removal in the dialyser, which is consistent with the literature [13, 14]. The results obtained in our studies using FTIR-ATR spectroscopy, however, revealed an increase in the amount of peroxidation products by 44% after 15 minutes, and 29% after 240 minutes of treatment, indicating the presence of oxidative stress in haemodialysis. This is also related to the amount of neutrophils and monocytes in the blood, which are responsible for the release of ROS. It was shown that the greatest decline in their numbers associated with activation and adhesion to the dialyser occurs in the first 15 minutes of dialysis [23, 36]. After this time, the number of cells gradually returns to baseline. Thus, the most intense release of ROS and lipid peroxidation occurs in the first minutes of haemodialysis, as confirmed by our results.

Measurements of primary products of peroxidation on isolated lipids were performed without the presence of proteins, which may also undergo oxidative processes. The tests found a full correlation with the determination of TBARS method. The usefulness of the proposed method for studying plasma lipid peroxidation during haemodialysis was confirmed.

## Figures and Tables

**Table 1:** Assignment of organic compounds to bands on the FTIR-ATR spectrum of plasma lipids; *ν*-stretching vibration, *δ*-bending vibrations, s-symmetric, as-asymmetric, and sh-shoulder band.

Wave number/cm^−1^		Assignment	Literature
On recorded spectra	According to literature		
3370	3280–3473	*ν*(O–H) and *ν*(N–H): water molecules, choline	[24, 25]
2958 sh	2952–2959 sh	*ν*_as_(CH_3_): lipids, cholesterol esters, fatty acids	[25–32]
2920	2919–2925	*ν*_as_(CH_2_): lipids, long-chain fatty acids	[25–32]
2868 sh	2871–2873 sh	*ν*_s_(CH_3_): lipids, fatty acids	[24, 25, 27]
2852	2850–2855	*ν*_s_(CH_2_): lipids, long-chain fatty acids	[25–32]
1738	1732–1747	*ν*(C=O): lipids, cholesterol esters, fatty acids oxides	[25, 27–30, 32]
1712	1712–1718	*ν*(C=O): COOH group of fatty acids	[26, 32]
1495 sh	1490 sh	*δ*_as_(N–CH_3_): choline group	[33]
1465	1464–1468	*δ*(CH_2_): aliphatic chains of fatty acids	[24–29]
1377	1377–1381	*δ*(CH_3_): aliphatic chains of fatty acids	[26–29]
1184	1160–1179	*ν*_as_(C–O): lipid ester bonds	[27, 28]
1082	1075–1090	*ν*_s_(PO_2_): phospholipids	[26, 27]
970	967–972	*ν*_as_(C=C): conformation *trans *	[26, 28, 29]
